# Supplementary material for: Transcript quantification using context-aware MAPQ modeling
Source: bioRxiv. 2026 Nov 1:2026.10.26.761703. Preprint. [Version 2] doi: 10.64898/2026.10.26.761703 (PMC13733865; doi:10.64898/2026.10.26.761703)
Supplement: Supplementary file 1 [file NIHPP2026.10.26.761703V2-supplement-1.pdf]

bioRxiv preprint doi: https://doi.org/10.64898/2026.10.26.761703; this version posted November 1, 2026. The copyright holder for this preprint (which was not certified by peer review) is the author/funder, who has granted bioRxiv a license to display the preprint in perpetuity. It is made available under a CC-BY 4.0 International license.

Supplementary Materials

Transcript quantification using context-aware MAPQ modeling

Sowmya Parthiban, Stephanie C. Hicks*

*Correspondence to shicks19@jhu.edu

Contents

1. **Supplementary Figures S1-S7**
2. **Supplementary Tables S1-S2**

bioRxiv preprint doi: https://doi.org/10.64898/2026.10.26.761703; this version posted November 1, 2026. The copyright holder for this preprint (which was not certified by peer review) is the author/funder, who has granted bioRxiv a license to display the preprint in perpetuity. It is made available under a CC-BY 4.0 International license.

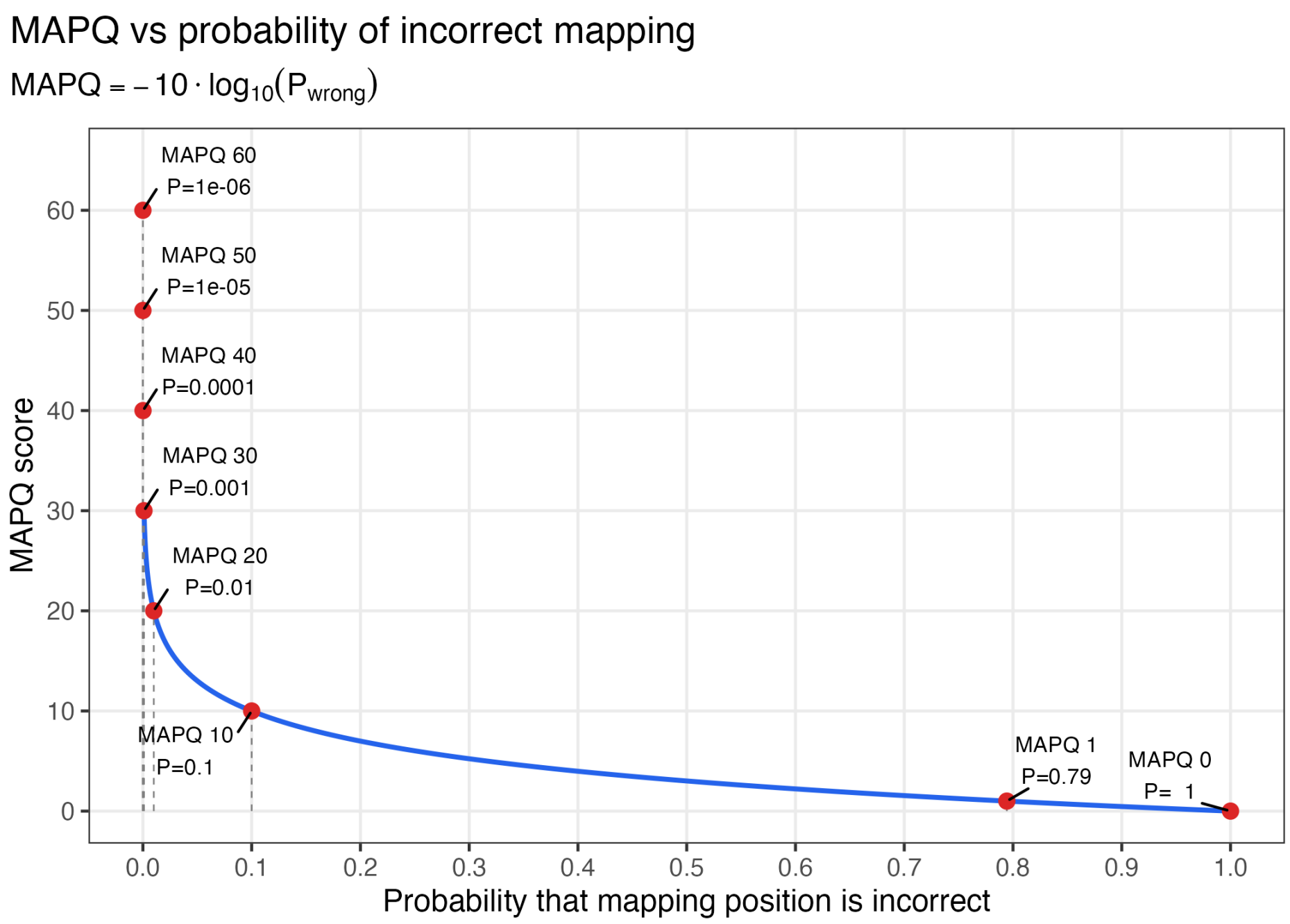

Supplementary Figure S1: Relationship between MAPQ score (y-axis) and the probability that a mapping position is incorrect, $\Pr\{\text{wrong}\} = 10^{-\text{MAPQ}/10}$ (x-axis). Red points mark MAPQ values 0, 1, 10, 20, 30, 40, 50, and 60, which are common thresholds to remove reads below a given value, such as a MAPQ of 30.

bioRxiv preprint doi: https://doi.org/10.64898/2026.10.26.761703; this version posted November 1, 2026. The copyright holder for this preprint (which was not certified by peer review) is the author/funder, who has granted bioRxiv a license to display the preprint in perpetuity. It is made available under a CC-BY 4.0 International license.

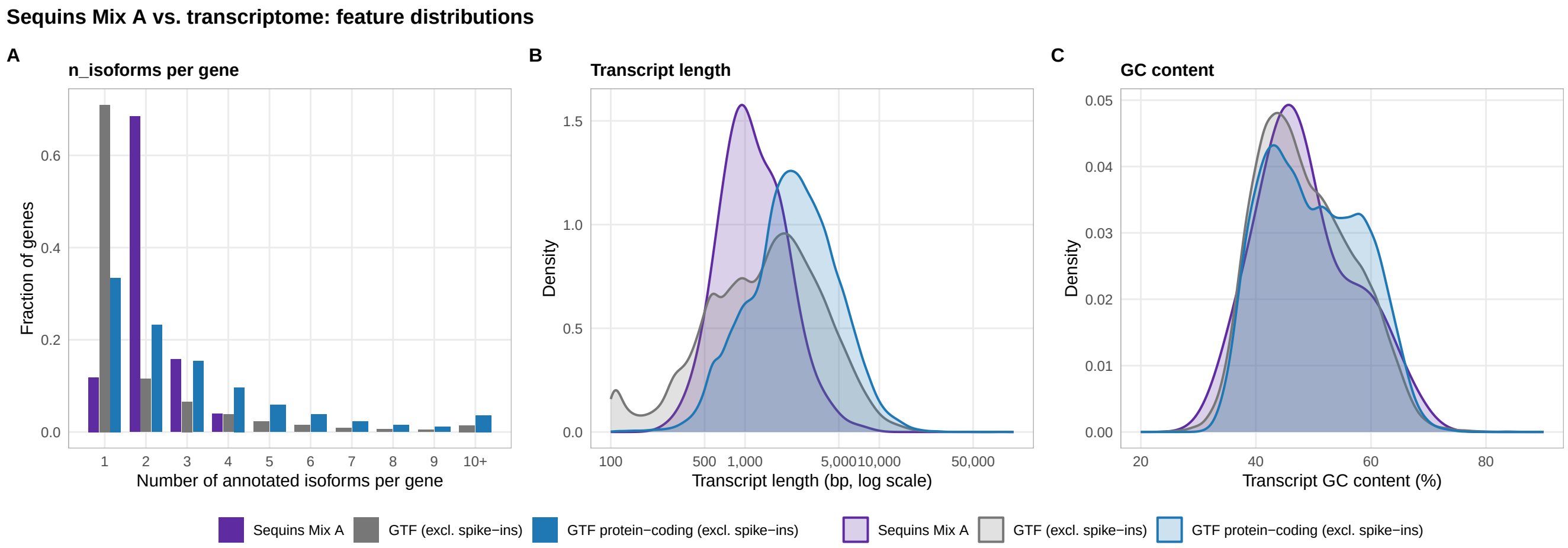

Supplementary Figure S2: Sequins Mix A feature distributions versus the transcriptome. **(A)** Overlapping per-category densities for number of annotated isoforms per gene, **(B)** transcript length (log scale), and **(C)** transcript GC content.

bioRxiv preprint doi: https://doi.org/10.64898/2026.10.26.761703; this version posted November 1, 2026. The copyright holder for this preprint (which was not certified by peer review) is the author/funder, who has granted bioRxiv a license to display the preprint in perpetuity. It is made available under a CC-BY 4.0 International license.

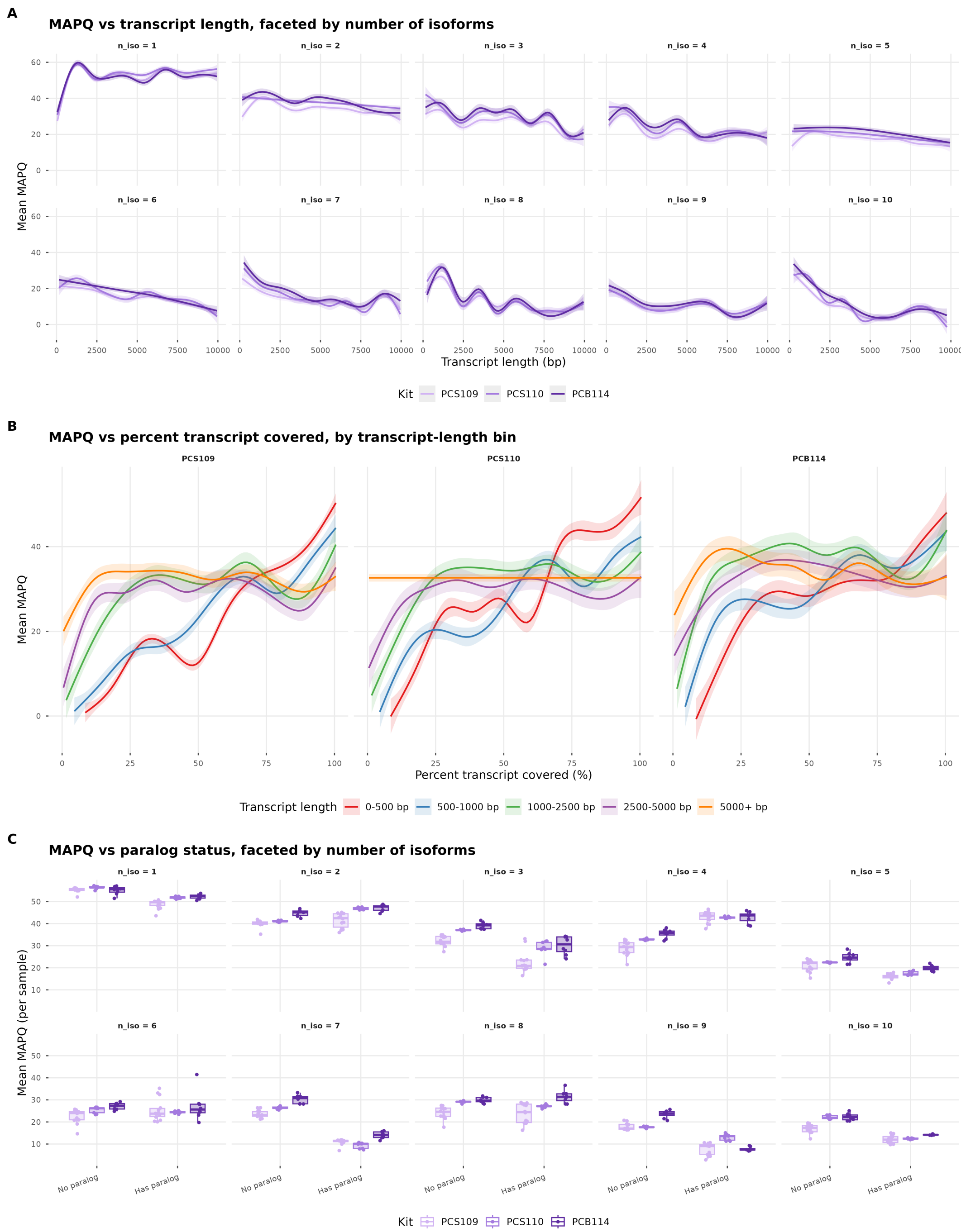

Supplementary Figure S3: The complex landscape of MAPQ between the interaction of isoform count, transcript coverage, transcript coverage, and paralog status. All panels use primary alignments from PCR cDNA kits PCS109, PCS110, and PCS114 and calculate per sample mean MAPQ for a given strata. (**A**) Mean MAPQ versus transcript length in 500 bp bins (up to 10,000 bp), stratified by annotated isoform count (1–10). Lines and ribbons show kit-specific means ±1 standard error across samples. (**B**) Mean MAPQ versus percent transcript covered (or fraction of reference transcript length), calculated at 1% resolution and averaged across samples, by kit and transcript-length class (0–500, 500–1000, 1000–2500, 2500–5000, 5000+ bp). Curves show smoothed Generalized Additive Models (GAM) with 95% confidence intervals. (**C**) Mean MAPQ for transcripts with or without cross-gene paralogs, stratified by isoform count. Boxplots summarize per-sample distributions (outliers suppressed); points are coloured by kit.

bioRxiv preprint doi: https://doi.org/10.64898/2026.10.26.761703; this version posted November 1, 2026. The copyright holder for this preprint (which was not certified by peer review) is the author/funder, who has granted bioRxiv a license to display the preprint in perpetuity. It is made available under a CC-BY 4.0 International license.

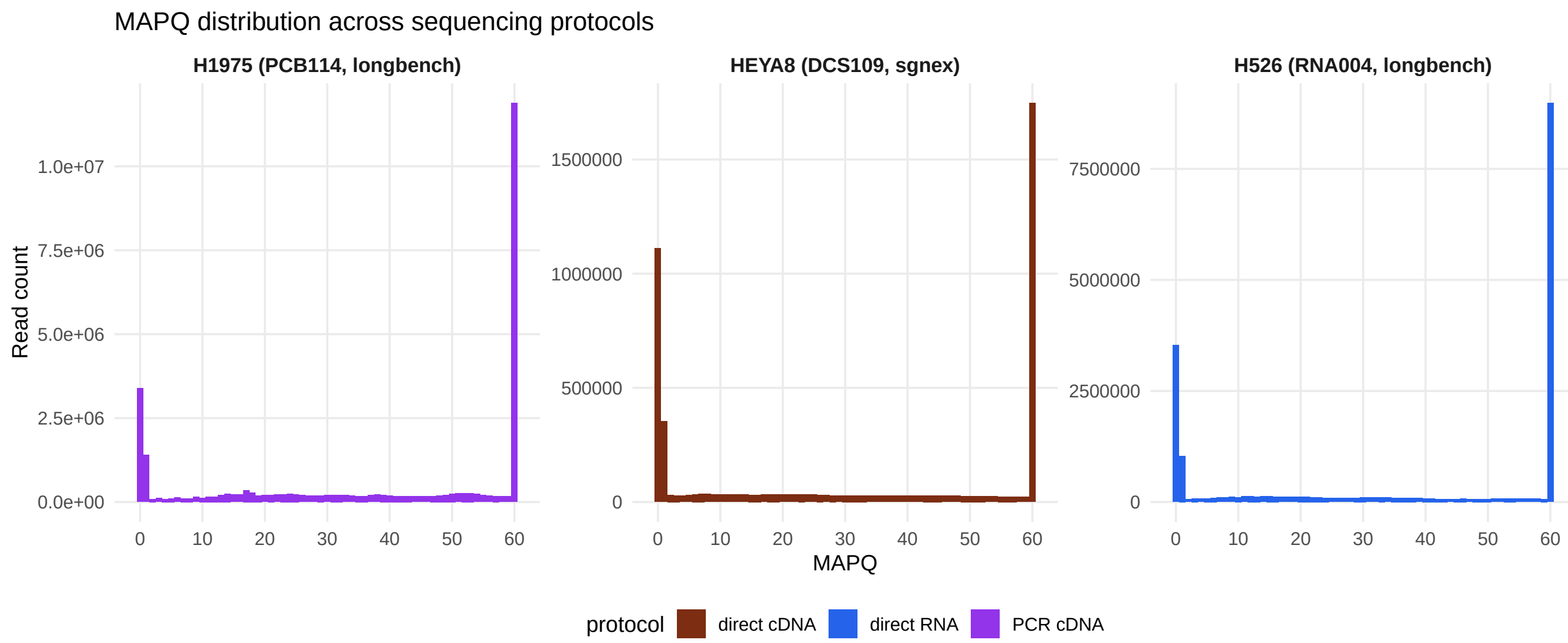

Supplementary Figure S4: MAPQ distribution varies with sequencing protocol. Per-read MAPQ histograms for one representative sample from each of three long-read sequencing protocols: **(A)** PCR cDNA, **(B)** direct RNA, and **(C)** direct cDNA. Each panel title lists the cell line, library-preparation kit, and source benchmark dataset for the displayed sample. Bars are coloured by protocol; the y-axis is read count and is free across panels because absolute read depth differs between samples. The characteristic bimodal shape (mass at MAPQ $= 0$ and MAPQ $= 60$) is present across all three chemistries.

bioRxiv preprint doi: https://doi.org/10.64898/2026.10.26.761703; this version posted November 1, 2026. The copyright holder for this preprint (which was not certified by peer review) is the author/funder, who has granted bioRxiv a license to display the preprint in perpetuity. It is made available under a CC-BY 4.0 International license.

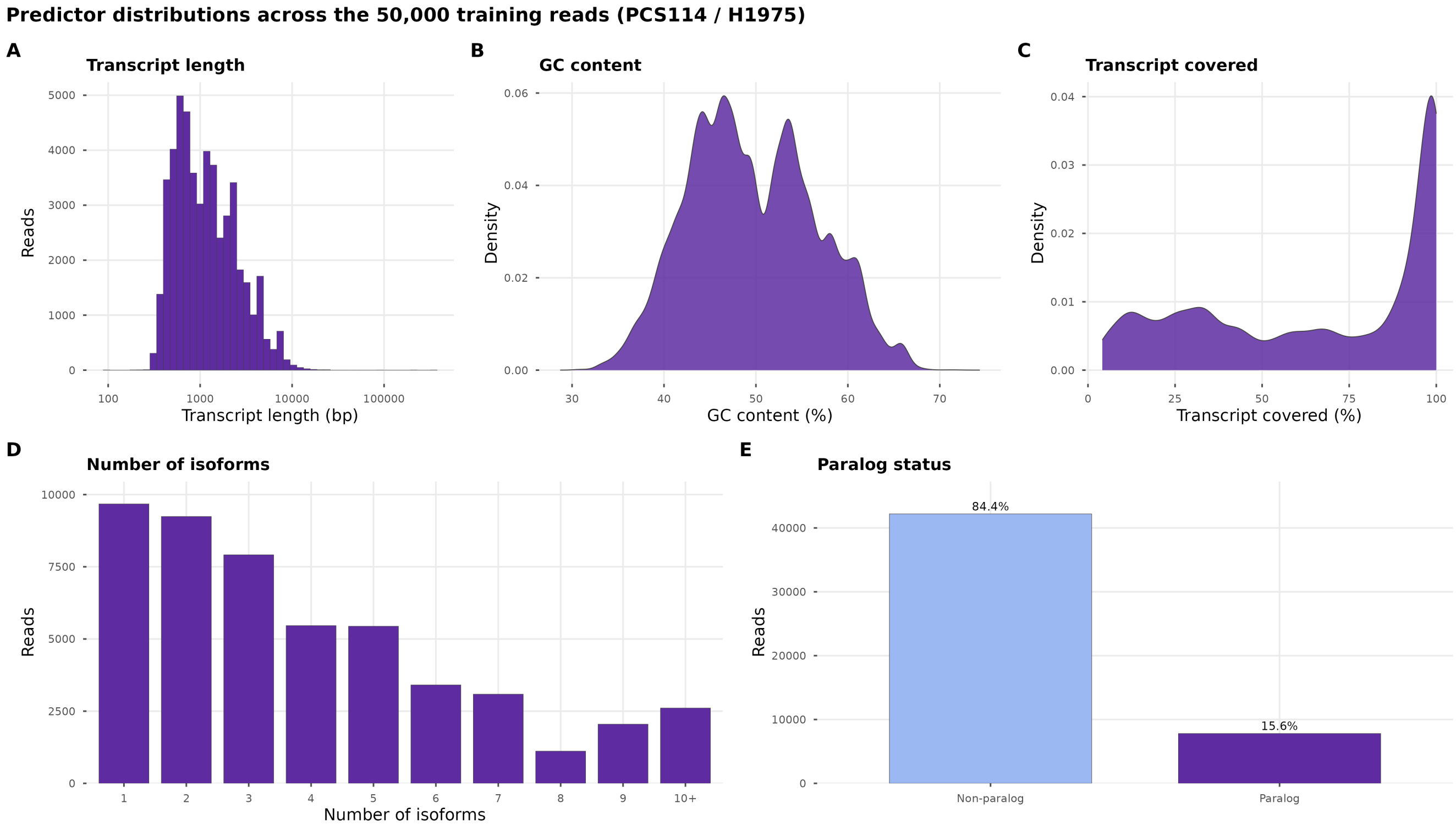

Supplementary Figure S5: Distributions of the five model predictors on the random subset of $n = 50{,}000$ primary alignments used to fit the BEINF model for the PCS114 / H1975 sample (GSM9135480). Values reflect the canonical preprocessing applied by `clean_features()`: **(A)** `n_isoforms` is lumped at 7 (counts ≥ 7 are clamped to 7); **(B)** `tx_length` is shown on a log-bp x-axis after the natural-log transform; **(C)** `gc_content` is plotted unchanged; **(D)** `pct_covered` is winsorized at the 1st and 99th percentiles of the training sample; **(E)** `has_paralog` is a binary indicator for at least one cross-gene paralog passing the alignment-stage filters. Histograms are used for the discrete or count-like predictors (`n_isoforms`, `tx_length`, `has_paralog`) and kernel density estimates for the continuous percentage variables (`gc_content`, `pct_covered`).

bioRxiv preprint doi: https://doi.org/10.64898/2026.10.26.761703; this version posted November 1, 2026. The copyright holder for this preprint (which was not certified by peer review) is the author/funder, who has granted bioRxiv a license to display the preprint in perpetuity. It is made available under a CC-BY 4.0 International license.

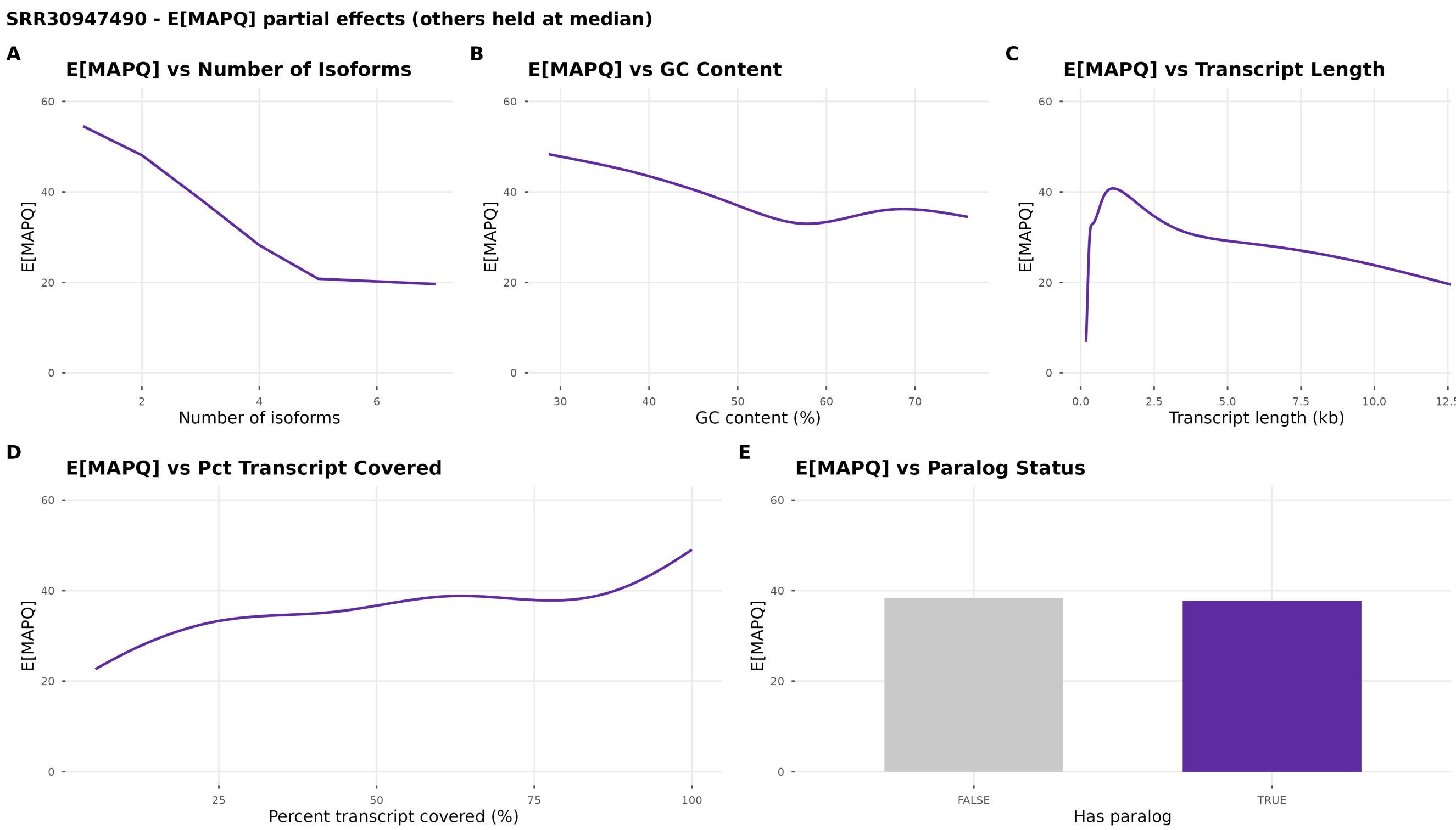

Supplementary Figure S6: Partial effects of each predictor on expected MAPQ, direct-RNA (RNA004). Direct-RNA counterpart of Figure 4, with the BEINF model refit on the RNA004 H1975 sample (Sequins Mix A; the direct-RNA analogue of the PCR H1975 sample). Each panel varies one predictor across its training range while holding the other predictors at their median value (or FALSE for the binary `has_paralog` indicator), and plots $\mathrm{E[MAPQ]} = 60\,(\tau + \mu)/(1 + \nu + \tau)$ under the resulting prediction. (A) `n_isoforms` is entered as a two-piece linear basis with a knot at 5. (B–D) The smoothed predictors `gc_content`, `tx_length` (log-bp x-axis), and `pct_covered` are entered through penalized B-splines and appear as continuous curves. (E) `has_paralog` is binary and shown as a column comparison. The partial-effect trends mirror those of the PCR cDNA model (Figure 4), indicating the MAPQ–feature relationships are not specific to the PCR preparation.

bioRxiv preprint doi: https://doi.org/10.64898/2026.10.26.761703; this version posted November 1, 2026. The copyright holder for this preprint (which was not certified by peer review) is the author/funder, who has granted bioRxiv a license to display the preprint in perpetuity. It is made available under a CC-BY 4.0 International license.

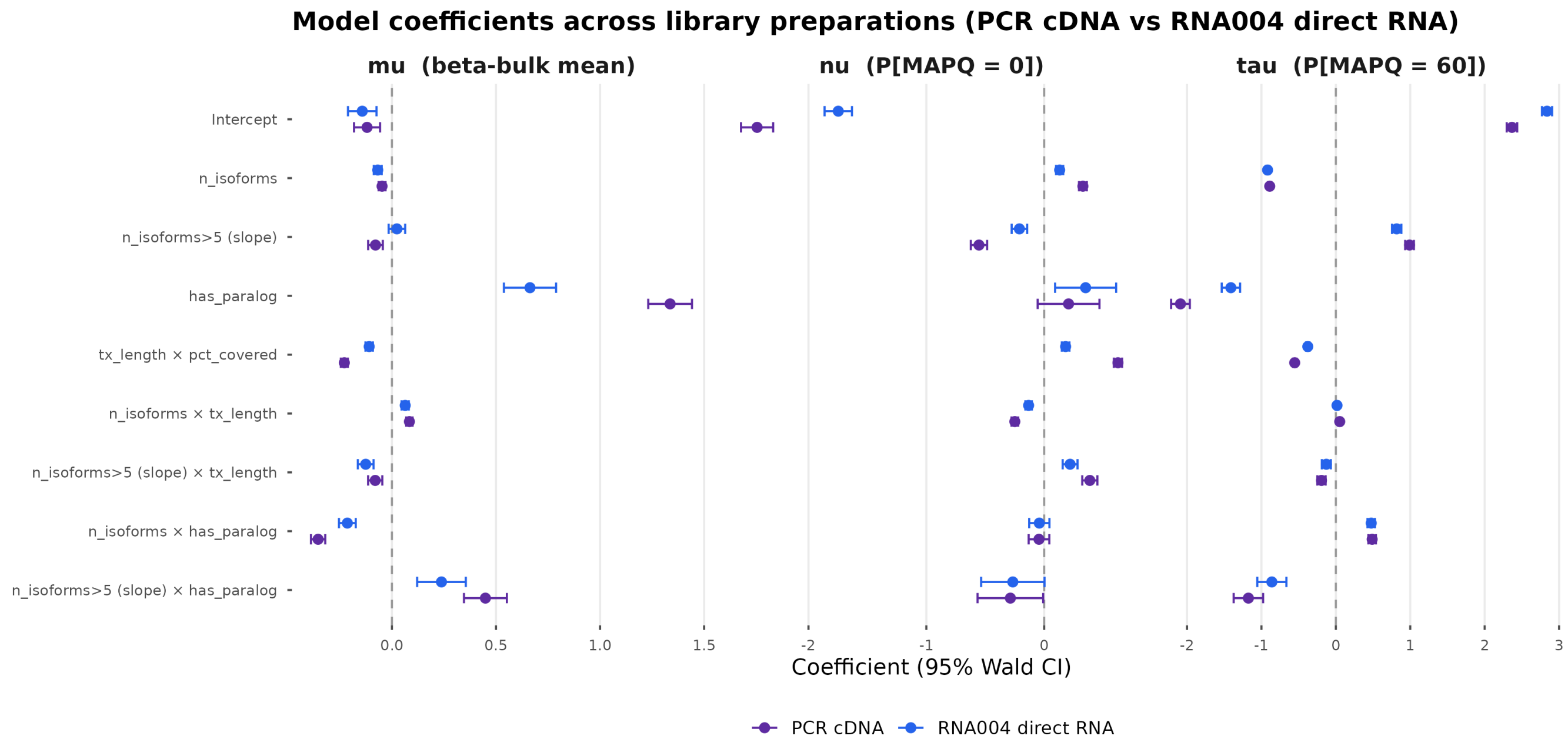

Supplementary Figure S7: Model coefficients are consistent across library preparations (PCR cDNA vs. RNA004 direct RNA). Parametric coefficients (points) with 95% Wald confidence intervals (horizontal bars) for the BEINF MAPQ model, overlaid for the two preparations of the H1975 cell line: PCR cDNA (`GSM9135480`, Sequins Mix A, purple) and RNA004 direct RNA (`SRR30947490`, blue). Both models use the same forward-stepwise, BIC-selected specification (penalized B-splines for the three continuous covariates, all five main effects, and all three interactions; μ, ν, and τ modeled, with $\sigma \sim 1$), refit independently on a 50,000 complete-case training subset of each sample. Panels correspond to the three modeled distribution parameters: μ (beta-bulk mean of MAPQ/60), ν (P[MAPQ = 0]), and τ (P[MAPQ = 60]). Penalized-spline basis terms are not interpretable point coefficients and are omitted; the displayed terms are the two-piece `n_isoforms` basis (with a knot at 5), `has_paralog`, the three interaction terms, and the intercept. The dashed vertical line marks a null coefficient of zero. The largely overlapping intervals between the two preparations indicate that the MAPQ–feature relationships learned by the model are not specific to the PCR protocol.

bioRxiv preprint doi: https://doi.org/10.64898/2026.10.26.761703; this version posted November 1, 2026. The copyright holder for this preprint (which was not certified by peer review) is the author/funder, who has granted bioRxiv a license to display the preprint in perpetuity. It is made available under a CC-BY 4.0 International license.

Supplementary Tables

Supplementary Table S1: Summary of spike-in datasets. Number of FASTQ files (runs) per protocol/kit combination across the five public benchmark datasets.

#	Protocol	Kit	CapTrap-seq	Dong et al.	LongBench	LRGASP	SG-NEx	Total
1	captrap	captrap_linker	2	0	0	6	0	8
2	PCR cDNA	PCS109	0	6	0	0	8	14
3	PCR cDNA	PCS110	0	0	0	9	0	9
4	PCR cDNA	PCS114	0	0	8	0	0	8
5	R2C2	R2C2	0	0	0	18	0	18
6	direct RNA	RNA002	2	0	0	12	14	28
7	direct RNA	RNA004	0	0	8	0	0	8
8	direct cDNA	DCS109	0	0	0	0	12	12
		**Total**	4	6	16	45	34	105

bioRxiv preprint doi: https://doi.org/10.64898/2026.10.26.761703; this version posted November 1, 2026. The copyright holder for this preprint (which was not certified by peer review) is the author/funder, who has granted bioRxiv a license to display the preprint in perpetuity. It is made available under a CC-BY 4.0 International license.

Supplementary Table S2: Spike-in datasets by benchmark, spike-in mix, protocol and kit. Counts are number of FASTQ files (runs).

**Benchmark**	**Spike-in mix**	**Protocol**	**Kit**	**n**
CapTrap-seq	ERCC, SIRV1 E1, SIRV1 E2	captrap	captrap_linker	2
CapTrap-seq	ERCC, SIRV1 E1, SIRV1 E2	direct RNA	RNA002	2
Dong et al.	Dong sequin Mix A	PCR cDNA	PCS109	3
Dong et al.	Dong sequin Mix B	PCR cDNA	PCS109	3
LongBench	Dong sequin Mix A , SIRV-4	PCR cDNA	PCS114	5
LongBench	Dong sequin Mix B , SIRV-4	PCR cDNA	PCS114	3
LongBench	Dong sequin Mix A , SIRV-4	direct RNA	RNA004	5
LongBench	Dong sequin Mix B , SIRV-4	direct RNA	RNA004	3
LRGASP	SIRV-4	captrap	captrap_linker	6
LRGASP	SIRV-4	PCR cDNA	PCS110	9
LRGASP	SIRV-4	R2C2	R2C2	18
LRGASP	SIRV-4	direct RNA	RNA002	12
SG-NEx	SGNex sequin Mix A V1	PCR cDNA	PCS109	2
SG-NEx	SIRV-4	PCR cDNA	PCS109	3
SG-NEx	SGNex sequin Mix A V2, SIRV-1 E2	PCR cDNA	PCS109	3
SG-NEx	SGNex sequin Mix A V1	direct cDNA	DCS109	2
SG-NEx	SGNex sequin Mix A V1	direct cDNA	DCS109	4
SG-NEx	SIRV-4	direct cDNA	DCS109	3
SG-NEx	SGNex sequin Mix A V2, SIRV-1 E2	direct cDNA	DCS109	3
SG-NEx	SGNex sequin Mix A V1	direct RNA	RNA002	4
SG-NEx	SIRV-4	direct RNA	RNA002	5
SG-NEx	SGNex sequin Mix A V2 , SIRV-1 E2	direct RNA	RNA002	5
			**Total**	**105**
